# Characterization of time‐dependent changes in the subchondral bone density distribution of the proximal tibia following high tibial osteotomy

**DOI:** 10.1002/jeo2.70517

**Published:** 2025-11-14

**Authors:** Yoshiaki Hosokawa, Koji Iwasaki, Taku Ebata, Dai Sato, Masanari Hamasaki, Masatake Matsuoka, Tomohiro Onodera, Eiji Kondo, Norimasa Iwasaki

**Affiliations:** ^1^ Department of Orthopaedic Surgery, Faculty of Medicine and Graduate School of Medicine Hokkaido University Sapporo Hokkaido Japan; ^2^ Department of Functional Reconstruction for the Knee Joint, Faculty of Medicine Hokkaido University Sapporo Hokkaido Japan; ^3^ Centre for Sports Medicine Hokkaido University Hospital Sapporo Hokkaido Japan

**Keywords:** CT‐osteoabsorptiometry, high tibial osteotomy, knee, osteoarthritis, subchondral bone density

## Abstract

**Purpose:**

This study aimed to evaluate the time‐dependent changes in the medial and lateral subchondral bone density distribution of the tibial joint surface before and after high tibial osteotomy (HTO) using computed tomography‐osteoabsorptiometry.

**Methods:**

This study included 17 patients (20 knees) (8 men and 9 women; mean age: 55 years) who underwent HTO for medial compartment osteoarthritis. Computed tomography‐osteoabsorptiometry was conducted to measure the subchondral bone density distribution in the tibial joint surface. The high‐density area (HDA), defined as the region corresponding to the highest Hounsfield unit values that comprises 20% of the total region, was calculated. Medial ratio was defined as the proportion of the HDA in the medial compartment relative to the total HDA of both compartments. Measurements were performed preoperatively and at 3, 6, 12 and 24 months post‐operatively, and a generalized linear regression analysis with a gamma distribution model was conducted.

**Results:**

The medial ratios (mean ± standard deviation) were 89 ± 9% preoperatively, 73 ± 12% at 3 months, 78 ± 13% at 6 months, 78 ± 10% at 12 months and 77 ± 17% at 24 months. Based on the gamma distribution, the non‐linear model was expressed as follows: *Y* = 82.8 × *t* ^−^^0.04^ × exp(−0.004 × *t*). This finding indicated that the medial ratio had a decreasing trend, reaching its minimum at 10.2 months post‐operatively.

**Conclusion:**

This study showed time‐dependent changes in the subchondral bone density on the tibial side after HTO. The changes in the bone density distribution on the medial and lateral tibial joint surface, which are associated with alignment correction, stabilized at approximately 10.5 months post‐operatively. Therefore, the optimal timing for evaluating subchondral bone density in response to stress redistribution may be after 10.2 months.

**Level of Evidence:**

Level IV.

**Abbreviations**
ACL: anterior cruciate ligament
CT: computed tomography
DEXA: dual‐energy X‐ray absorptiometry
HKA: hip–knee–ankle angle
HTO: high tibial osteotomy
HU: Hounsfield unit
iV‐HTO: inverted‐V‐shaped HTO
KL: Kellgren–Lawrence
mLDFA: mechanical lateral distal femoral angle
MMPRT: medial meniscus posterior root tear
mMPTA: mechanical medial proximal tibial angle
OA: osteoarthritis
OAT: osteochondral autograft transplantation
PF: patellofemoral

## INTRODUCTION

High tibial osteotomy (HTO) is an effective joint‐preserving procedure established for treating medial knee osteoarthritis (OA) and spontaneous osteonecrosis of the knee [14, 20]. HTO alleviates pain by reducing the load of the medial compartment via valgus alignment correction of the lower limb. However, evaluating the stress distribution within the living body directly and invasively remains challenging. Computed tomography (CT)‐osteoabsorptiometry was developed to estimate the stress distribution on articular surfaces in vivo by measuring the bone density of the subchondral bone using CT scan [17]. This method facilitates the analysis of long‐term stress distribution on joints in vivo via CT scan, which is a minimally invasive procedure. Several studies have estimated the stress distribution in various joints using CT‐osteoabsorptiometry [6, 8, 9, 15, 16].

Previous studies revealed that mediolateral ratio of high‐density area (HDA) of subchondral bone across the proximal tibia (medial ratio) was correlated with leg alignment in patients with medial knee OA and that the medial ratio decreased after HTO, dependent on the change in the leg alignment before and 1.5 years after HTO [4, 7]. However, the time‐dependent changes in subchondral bone density resulting from abrupt stress redistribution, such as that caused by alignment correction through HTO or by medial meniscus posterior root tear (MMPRT), have not been fully elucidated. It also remains unclear whether the timing of evaluation at 1.5 years post‐operatively in previous studies [4, 7] was appropriate. Without clarifying these time‐dependent changes, the optimal timing for evaluating subchondral bone density using CT‐osteoabsorptiometry remains uncertain. Therefore, the current study aimed to elucidate the characterization of time‐dependent changes in subchondral bone density distribution on the medial and lateral aspects of the proximal tibial joint surface before and after HTO using CT‐osteoabsorptiometry. The hypothesis of this study was that, following an abrupt change in mechanical stress such as that induced by HTO, the distribution of subchondral bone density would stabilize within 1.5 years post‐operatively—the time point at which previous studies conducted their evaluations.

## METHODS

### Study design

This clinical study was retrospectively performed at Hokkaido University Hospital and was approved by the institutional review board of Hokkaido University Hospital (No.: 018‐0213). This study included patients with medial compartment knee OA who underwent HTO around the knee joint at our institution between 2016 and 2021. All surgeries were performed by senior orthopaedic surgeons (EK and KI) who had >10 years of experience in performing knee surgery. We performed two types of HTO: inverted‐V‐shaped HTO (iV‐HTO) and open‐wedge HTO (OWHTO). The inclusion criteria for iV‐HTO were as follows: (1) a knee requiring a valgus correction of ≥10° to achieve the post‐operative lower leg alignment of the mechanical axis of 65% or (2) a knee with Kellgren–Lawrence (KL) Grade 2 or higher patellofemoral (PF) OA [10]. iV‐HTO is selected for patients with PF OA, as it improves patellofemoral joint congruency and is less likely to negatively affect the patellofemoral joint [11]. The inclusion criterion for OWHTO was a knee requiring less than 10° of correction and with PF OA of KL Grade 1 or lower. Cases with mechanical lateral distal femoral angle (mLDFA) greater than 92° or (2) planned post‐operative mechanical medial proximal tibial angle (mMPTA) following HTO exceeded 97° were indicated for double‐level osteotomy. At our institution, CT scan was performed at 3, 6, 12 and 24 months post‐operatively to evaluate bone union and changes in subchondral bone density after osteotomy until 2022.

The exclusion criteria were as follows: (1) cases in which CT evaluation could not be available at the designated time points or where the timing deviated by more than 1 month for the 3‐ and 6‐month evaluations or by more than 2 months for the 12‐ and 24‐month evaluations, (2) cases in which screws were placed within 10 mm of the joint surface, based on the fact that titanium fixation devices typically generate artefacts extending 1–3 mm [19], (3) cases of under correction (final hip–knee–ankle angle [HKA] more varus than 1° valgus) or pseudoarthrosis, (4) cases with compartment syndrome, which prevented adherence to the post‐operative weight‐bearing protocol and (5) cases with simultaneous MMPRT repair or autologous osteochondral transplantation.

### Surgical procedure

The iV‐HTO is classified as a neutral wedge osteotomy (hemiclosing and hemiopening) that requires a concomitant fibular osteotomy [2]. The locking plate (TomoFix Lateral High Tibia Plate [DePuy Synthes] or Tris Lateral Tibia Plate [Olympus Terumo Biomaterials]) was fixed on the lateral side of the tibia. Finally, the resected bone block was implanted in the medial opening space [11, 12]. In the OWHTO procedure, an ascending biplanar osteotomy of the tibial tubercle was performed, and wedge‐shaped β‐tricalcium phosphate spacers (Osferion 60; Olympus Terumo Biomaterials) were implanted into the opening gap. The tibia was then fixed with a locking plate (TriS Medial HTO plate system, Olympus Terumo Biomaterials) [9]. In iV‐HTO, partial weight‐bearing on the tibia was permitted with crutches 2 weeks after surgery, and patients remained hospitalized until full weight‐bearing was allowed at 4 weeks post‐operatively. In OWHTO, full weight‐bearing was permitted 1 week after surgery, and patients were discharged once they could ambulate safely without crutches. All patients were able to ambulate without crutches upon discharge within 4 weeks.

### Radiographic evaluation

**Figure 1:**
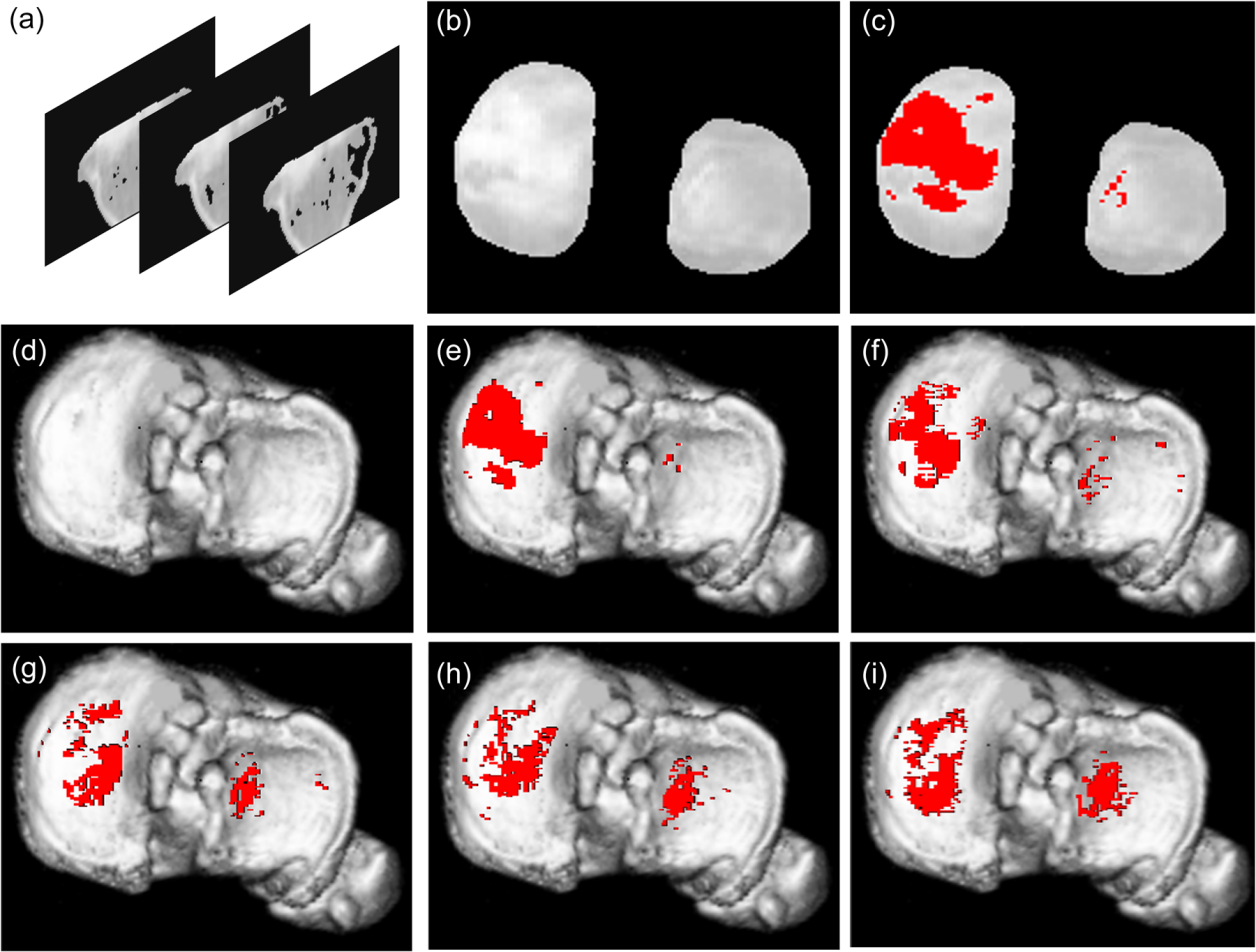
Time‐dependent changes in subchondral bone density in a representative case following high tibial osteotomy (HTO). (a) Subchondral bone density of the selected region was automatically quantified at each coordinate point in every 1.0‐mm sagittal slice. (b) The medial and lateral articular surfaces were extracted from the stacked images. (c) Areas highlighted in red indicate high‐density area. (d) Three‐dimensional reconstruction of the proximal tibial articular surface. (e) Representative changes in the high‐density area: preoperative image with the three‐dimensional reconstruction merged with the high‐density area; (f) 3 months after HTO; (g) 6 months; (h) 12 months; and (i) 24 months.

HKA, mMPTA and mLDFA were assessed using full‐length standing radiographs taken preoperatively and at 1 year post‐operatively [6, 11]. Radiographic measurements were performed by YH, an orthopaedic surgeon with 15 years of clinical experience. A high‐resolution helical CT scanner (Aquilion One/ViSION Edition; Toshiba Medical Systems) was used to acquire axial images of the knee. The slice thickness and interval were set at 0.5 mm. CT scan procedures were performed preoperatively and at 3, 6, 12 and 24 months post‐operatively. CT‐osteoabsorptiometry was conducted in accordance with previous reports [4, 7]. Sagittal slices at 1.0‐mm intervals and three‐dimensional bone models were generated from axial CT scan data using commercial software (Ziocube®; Ziosoft, Inc.). Then, the highest CT scan value (Hounsfield unit, HU) of the subchondral bone density within 3.0 mm from the articular surface of each generated sagittal slice was analyzed with a noncommercial software (OsteoDens 4.0) that was developed at our institution [4, 7, 9, 15, 16]. A distribution map of the subchondral bone HU values on the knee joint surface was obtained by stacking sagittal slices containing the HU value data (Figure 1a). HDA was defined as the region corresponding to the highest HU values that comprises 20% of the total region of the medial and lateral compartments (Figure 1b,c). This relative measure was used to account for interindividual differences in absolute HU values related to body weight, age and sex, allowing standardized relative comparisons across patients. The medial ratio was calculated as the ratio of the HDA of the medial compartment to the total HDA of both compartments. We analyzed the medial ratio preoperatively and at 3, 6, 12 and 24 months post‐operatively (Figures 1d–i).

### Statistical analysis

Statistical analyses were performed using JMP Pro 14.0 (SAS Institute Inc.). A *p* value of 0.05 indicated statistically significant differences. Repeated measures analysis of variance was performed to evaluate time‐dependent changes, followed by Dunnett's post hoc test to compare preoperative values with post‐operative values. Association between the change in medial ratio and HKA before and 1 year after HTO was evaluated by using Pearson's correlation analysis. To evaluate time‐dependent changes in the medial ratio, non‐linear regression models based on a gamma‐type function were applied using custom scripts in MATLAB® (The MathWorks, Inc.). A random effect structure was incorporated using a linear mixed‐effects model, treating individual patient differences as random effects. The gamma‐type model, defined as: *y*(*t*) = *a* · *t* ^*b*^ · exp(−*c* · *t*), allowed for flexible capture of the asymmetric and unimodal temporal behaviour of subchondral bone remodelling without imposing assumptions about the timing or shape of the curve.

Despite the relatively small number of subjects (*n* = 20), the model demonstrated a high adjusted *R* ^2^ (0.761) with a gamma‐type time function and random patient effects. The effect size (*f* ^2^ = 3.18) was large, indicating that the model captured substantial variance in the outcome. With three degrees of freedom for the fixed effects, the statistical power was estimated to be high (>0.90), although this estimate should be interpreted with caution, given the small sample size.

Using radiographs from 10 cases, the intraobserver and interobserver reliability of HKA measurements was assessed by the examiner (YH and TE) with a 1‐week interval between measurements, resulting in the intraobserver and interobserver intraclass correlation coefficients of 0.951 and 0.943, respectively. A previous study reported high reproducibility in evaluating the medial ratio using the noncommercial software OsteoDens 4.0. The intraclass correlation coefficients for intraobserver reliability were 0.900 and 0.920. The intraclass correlation coefficient for interobserver reproducibility was 0.910 [4].

## RESULTS

**Figure 2:**
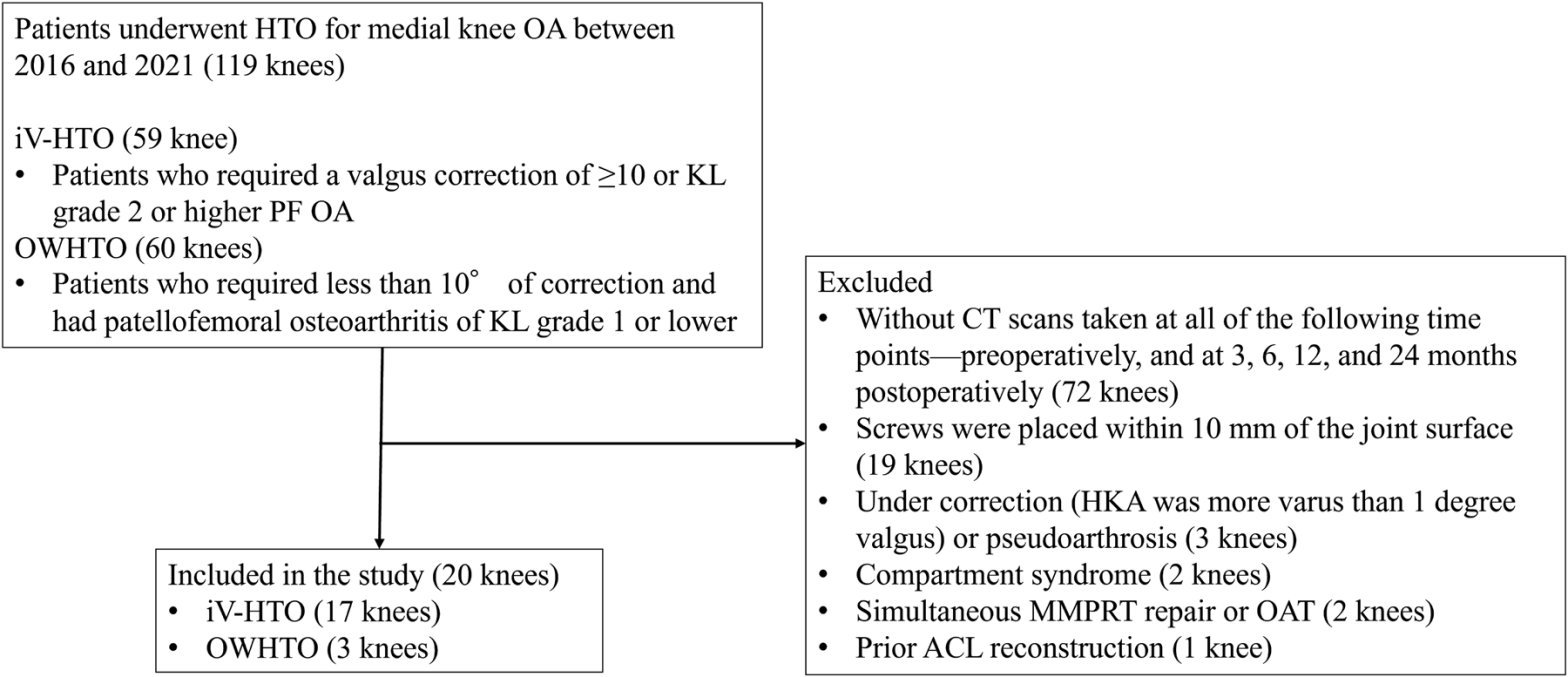
Flowchart of study enrolment. ACL, anterior cruciate ligament; DLO, double‐level osteotomy; HTO, high tibial osteotomy; iV‐HTO, inverted‐V shaped high tibial osteotomy; KL, Kellgren–Lawrence; mLDFA, mechanical lateral distal femoral angle; MMPRT, medial meniscus posterior root tear; mMPTA, mechanical medial proximal tibial angle; OA, osteoarthritis; OAT, osteochondral autograft transplantation; OWHTO, open wedge HTO; PF, patellofemoral.

After applying the exclusion criteria (with some cases meeting multiple criteria), 20 knees (17 patients, with 8 men and 9 women) were finally evaluated (Figure 2). All knees had complete CT data available preoperatively and at 3, 6, 12 and 24 months post‐operatively. The average age of the patients was 54 ± 9 years; the average height, 156 ± 10 cm; the average weight, 69 ± 14 kg; the average body mass index, 28 ± 4 kg/m^2^; and smoking, 3 patients. KL grade of tibiofemoral joint, Grade 2 in 5 knees, Grade 3 in 12 and Grade 4 in 3. In the radiographic evaluation, HKA changed significantly from −7 ± 4° preoperatively to 3 ± 4° post‐operatively (*p* < 0.01). Further, MPTA changed significantly from 83 ± 3° preoperatively to 92 ± 4° post‐operatively (*p* < 0.01) (Table 1). Simultaneous medial partial meniscectomy was performed in three knees.

**Table 1:** Patient characteristics before and after HTO.

	Preoperative value	Post‐operative value	*p*
Age, years	56 ± 9	n/a	n/a
Male:female ratio (*n*)	8:9	n/a	n/a
KL grades (II, III and IV) (*n*)	5: 12: 3	5: 12: 3	N.S.
HKA, degrees	−7 ± 4	3 ± 4	<0.01
MPTA, degrees	83 ± 3	92 ± 4	<0.01

*Note*: Data are expressed as mean ± SD.

Abbreviations: HKA, hip–knee–ankle angle; HTO, high tibial osteotomy; KL grade, Kellgren–Lawrence grade; MPTA, medial proximal tibial angle; N.S., not significant.

**Figure 3:**
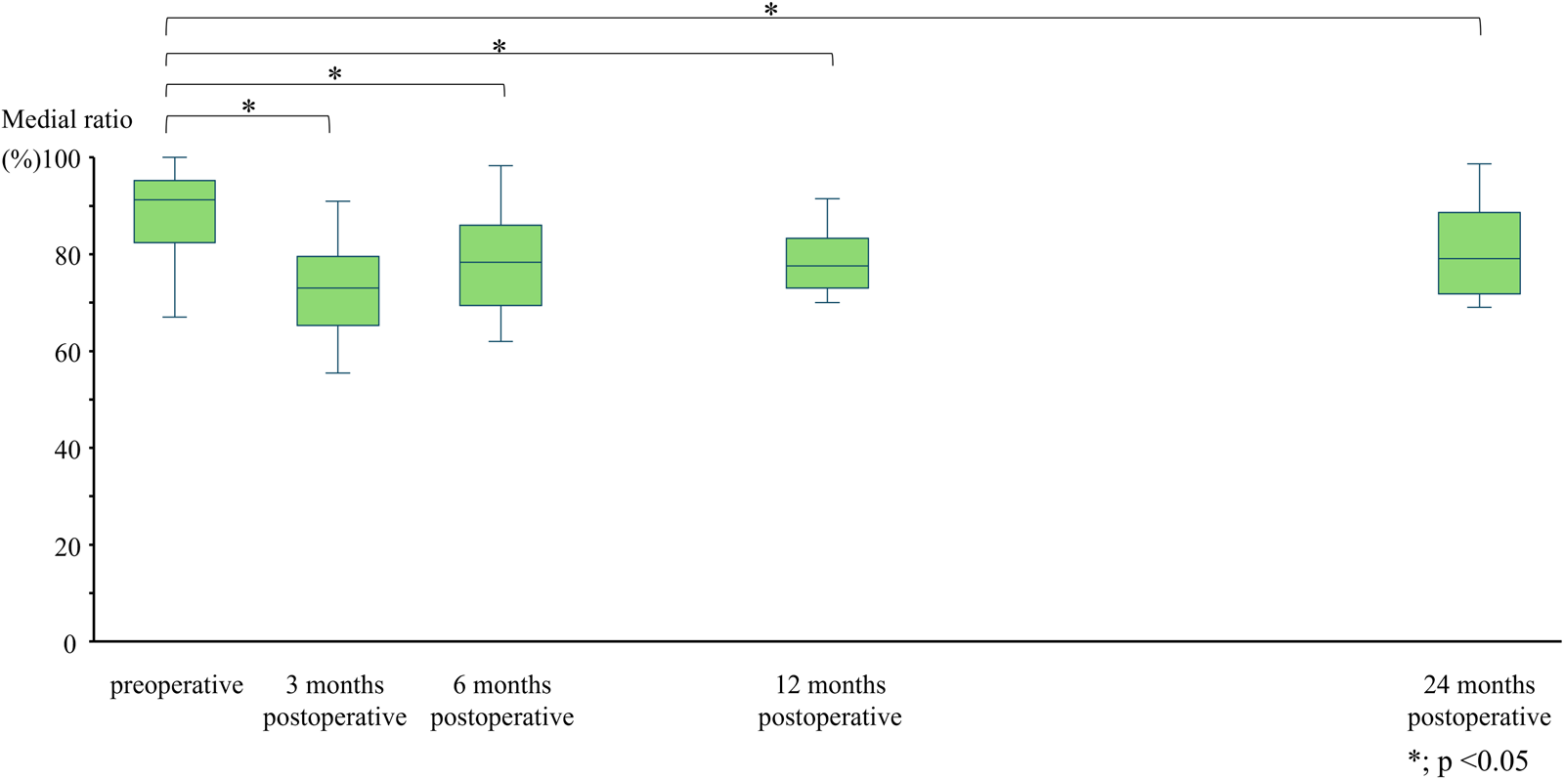
The temporal changes in the medial ratio. Preoperative 89 ± 9%, post‐operative 3 months 73 ± 12%, post‐operative 6 months 78 ± 13%, post‐operative 12 months 78 ± 10% and post‐operative 24 months 77 ± 17%.

**Figure 4:**
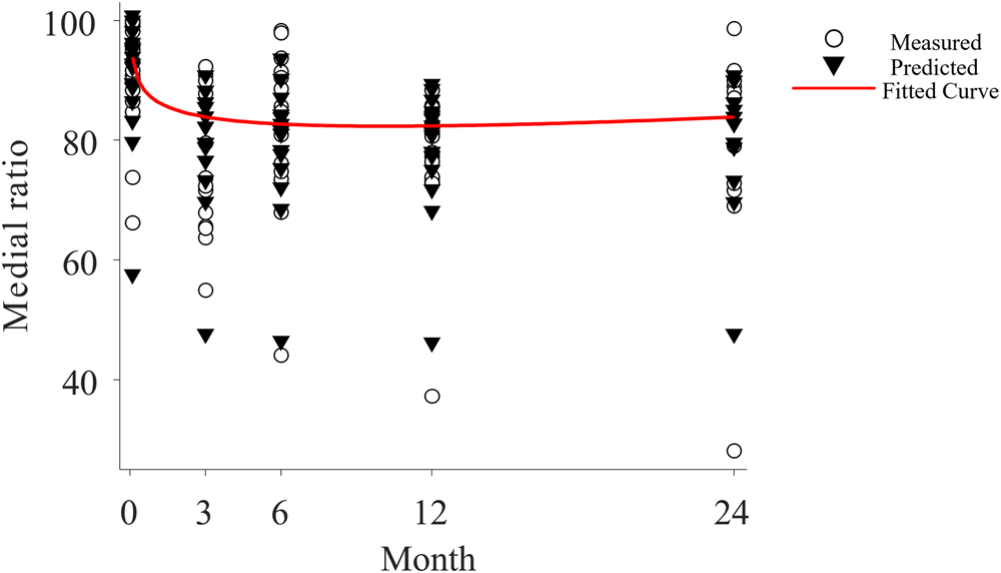
Time‐dependent changes in the medial ratio after high tibial osteotomy, analyzed using a non‐linear regression model based on a gamma‐type function. Open circles represent observed values at each time point (preoperatively, and at 3, 6, 12 and 24 months post‐operatively), while filled inverted triangles indicate predicted values derived from the model. The red line represents the fitted curve based on the gamma distribution model: *Y* = 82.8 × *t* ^−0.04^ × exp(−0.004 × *t*), capturing the estimated trajectory over time.

The time‐dependent changes in the medial ratio were as follows: 89 ± 9% preoperatively, 73 ± 12% at 3 months post‐operatively, 78 ± 13% at 6 months, 78 ± 10% at 12 months and 77 ± 17% at 24 months (Figure 3). The post‐operative values at all time points significantly decreased compared with the preoperative values (Figure 3). The change in HKA was significantly correlated to the change in medial ratio (*r* = −0.52, *p* = 0.04) before and 1 year after HTO. Based on the gamma‐type function, the non‐linear model was expressed as follows: *Y* = 82.8 × *t* ^−0.04^ × exp(−0.004 × *t*). An evaluation of the model's fit showed that the root mean square error was 7.2. Moreover, the coefficient of determination (*R* ^2^) was 0.762. This finding indicated that the medial ratio exhibited a decreasing trend, reaching its minimum at 10.2 months post‐operatively (Figure 4).

## DISCUSSION

The main findings of the present study were that subchondral bone density decreased 3 months after HTO and tended to stabilize at 10 months post‐operatively.

A previous study on bone density changes after HTO using DEXA (dual‐energy X‐ray absorptiometry) of the femoral medial and lateral condyles demonstrated that the medial‐to‐lateral ratio decreased at 3, 6 and 12 months post‐operatively. However, significant changes were not observed after 12 months [1]. In this study, the subchondral bone of the proximal tibial joint surface was evaluated using CT‐ osteoabsorptiometry. The medial ratio showed a significant decrease at 3 months post‐operatively. However, the regression analysis revealed that the decreasing trend plateaued at 10 months. Compared with a previous report [1], this study found that the medial‐to‐lateral ratio plateaued slightly earlier. This discrepancy could be explained by the use of different evaluation methods, including DEXA, which is used to assess both the trabecular and cortical bone, and CT‐osteoabsorptiometry, which is utilized to evaluate the subchondral bone alone. In addition, the bone turnover in the subchondral bone is faster than that in the trabecular bone [5], which might have also influenced the results. The results of the current study suggested that a time point beyond 10 months might be appropriate for evaluating bone density changes associated with stress distribution alterations after HTO.

Bone metabolic turnover comprises bone formation and turnover, and mechanical stimulation is one of the most important drivers of bone metabolism [13]. According to Frost's theory, if the mechanical stress on the bone tissue decreases, it falls below the resorption threshold, thereby disrupting the supply of nutrients to the bone tissue and triggering apoptosis [3]. As a result, osteoclasts are activated, leading to bone resorption, and the bone density decreases. It is considered that the change in stress distribution from the early post‐operative period led to a shift in bone metabolic turnover, resulting in a decrease in the medial ratio. By contrast, the study results showed that the medial ratio decreased until 10 months post‐operatively. This finding suggested that the timing of evaluating subchondral bone density distribution should be approached with caution in cases of rapid changes in joint stress distribution, such as after osteotomy.

A study comparing the tibial and radial bone density before and after a 1‐year stay in space reported a higher rate of bone density decrease in the tibia [21]. This result indicated that bones that are under greater load are more affected by unloading, leading to a larger impact on bone density. In this study, which compared 3 months post‐operatively with later time points, the medial ratio was more likely to be lower at 3 months post‐operatively. Based on this finding, due to unloading after surgery, the bone density in both the medial and lateral sides decreased. However, the bone density in the medial side, which was originally under greater load, was more likely to decrease. After 3 months post‐operatively, the influence of unloading might have decreased, and the bone density could have slightly increased.

CT‐osteoabsorptiometry is a technique used to identify regions on the joint surface that are subject to high mechanical stress, as well as to evaluate how these regions change in response to interventions such as HTO. In previous studies, we divided the articular surface into eight medial‐to‐lateral regions and found that the area near the intercondylar eminence on the medial compartment showed increased density following HTO, which was associated with post‐operative HKA angle and MPTA [4]. Furthermore, we observed a positive correlation between the time from anterior cruciate ligament injury to CT imaging and the density in the posterior region of the medial compartment, when dividing the medial compartment into three anterior‐posterior sections [16]. Therefore, this method was considered a reasonable approach for analyzing changes in high‐density regions of subchondral bone.

Several limitations of this study should be acknowledged. First, the statistical analysis was based on a non‐linear model using a gamma‐type time function. This model demonstrated a high adjusted R² (0.761) with random patient effects, indicating a good overall fit and supporting the appropriateness of the chosen model. The effect size was large (*f* ^2^ = 3.18), and the post hoc statistical power, calculated with three degrees of freedom for the fixed effects, was estimated to be high (>0.90). Nevertheless, this estimate should be interpreted with caution, given the small sample size. Furthermore, the validity of these results depends on the assumption that the gamma‐type function adequately characterizes the underlying time‐dependent behaviour. Second, the study population included all eligible HTO cases, but the cohort predominantly consisted of iV‐HTO, with only three OWHTO cases. Because iV‐HTO is typically indicated for knees requiring larger correction angles, this composition may still introduce a degree of selection bias. Moreover, important procedural differences exist between iV‐HTO and OWHTO, such as the timing of post‐operative weight‐bearing (full weight‐bearing permitted at 4 weeks after iV‐HTO vs. 1 week after OWHTO) and the concomitant performance of fibular osteotomy, which alone has been reported to influence load distribution [18]. Thus, while the present findings reflect outcomes after HTO, they should be interpreted with caution when applying these findings to HTO in general. Third, the subchondral bone density on the tibial side was evaluated regardless of screw removal. Because it was necessary to perform evaluations while the fixation screws were still in place, when assessing time‐dependent changes on the tibial side. However, since titanium artefacts are generally confined to approximately 1–3 mm [19], the analysis was limited to within 3 mm from the articular surface, and cases in which screws were located within 10 mm of the joint surface were excluded from the analysis. Therefore, the influence of metal artefacts was considered minimal. Fourth, there was no sensitivity analysis to assess how small changes in HU thresholds or segmentation might affect the results. Therefore, the findings of this study are based on the specific conditions defined by previous research (the top 20% HDA threshold and a 3‐mm evaluation depth), and it should be recognized that the results may not be generalizable if these conditions are altered.

## CONCLUSION

This study evaluated the time‐dependent changes in the subchondral bone density after HTO. The changes in the bone density distribution on the medial and lateral sides of the knee joint after HTO were found to have a decreasing trend up to 10 months post‐operatively.

## AUTHOR CONTRIBUTIONS

Yoshiaki Hosokawa and Koji Iwasaki conceived the project and designed the experiments. Yoshiaki Hosokawa, Koji Iwasaki, Taku Ebata, Dai Sato and Masanari Hamasaki collected and assembled data. Eiji Kondo and Koji Iwasaki recruited patients. Yoshiaki Hosokawa and Koji Iwasaki prepared the draft of the manuscript. Koji Iwasaki secured funding for the project. Dai Sato, Masatake Matsuoka, Tomohiro Onodera, Eiji Kondo and Norimasa Iwasaki supervised the project. All authors critically reviewed the manuscript and provided their comments.

## CONFLICT OF INTEREST STATEMENT

The institution of an author (Koji Iwasaki) has received funding from Olympus Terumo Biomaterials CORP. The remaining authors declare no conflicts of interest.

## ETHICS STATEMENT

The Institutional Review Board of Hokkaido University Hospital (No. #018‐0213).

## Data Availability

The authors elect not to share data.
